# Signal regulatory protein α associated with the progression of oral leukoplakia and oral squamous cell carcinoma regulates phenotype switch of macrophages

**DOI:** 10.18632/oncotarget.12874

**Published:** 2016-10-25

**Authors:** Xiaojing Ye, Jing Zhang, Rui Lu, Gang Zhou

**Affiliations:** ^1^ The State Key Laboratory Breeding Base of Basic Science of Stomatology (Hubei-MOST) and Key Laboratory of Oral Biomedicine Ministry of Education, School and Hospital of Stomatology, Wuhan University, Wuhan, P.R. China; ^2^ Department of Oral Medicine, School and Hospital of Stomatology, Wuhan University, Wuhan, P.R. China

**Keywords:** signal regulatory protein α, macrophage, oral leukoplakia, oral squamous cell carcinoma

## Abstract

Signal regulatory protein α (SIRPα) is a cell-surface protein expressed on macrophages that are *reg*arded as an important component of the tumor microenvironment. The expression of SIRPα in oral leukoplakia (OLK) and oral squamous cell carcinoma (OSCC), and further explored the role of SIRPα on the phenotype, phagocytosis ability, migration, and invasion of macrophages in OSCC were investigated. The expression of SIRPα in OLK was higher than in OSCC, correlating with the expression of CD68 and CD163 on macrophages. After cultured with the conditioned media of oral cancer cells, the expression of SIRPα on THP-1 cells was decreased gradually. In co-culture system, macrophages were induced into M2 phenotype by oral cancer cells. Blockade of SIRPα inhibited phagocytosis ability and IL-6, TNF-α productions of macrophages. In addition, the proliferation, migration, and IL-10, TGF-β productions of macrophages were upregulated after blockade of SIRPα. Macrophages upregulated the expression of SIRPα and phagocytosis ability, and inhibited the migration and invasion when the activation of NF-κB was inhibited by pyrrolidine dithiocarbamate ammonium (PDTC). Hence, SIRPα might play an important role in the progression of OLK and oral cancer, and could be a pivotal therapeutic target in OSCC by regulating the phenotype of macrophages via targeting NF-κB.

## INTRODUCTION

As one of the most common cancers in the world, oral squamous cell carcinoma (OSCC) is characterized by high cervical lymph node metastasis and poor prognosis [1, 2]. The development from oral precancer to oral cancer in histopathology level may undergo epithelial hyperplasia, dysplasia, early invasive stage as well as lymph node metastasis [3]. Recently, the tumor microenvironment has been known as an important hallmark of cancer [4]. In tumor microenvironments, macrophages are characterized as a major inflammatory component of the stroma and affect many aspects of the tumor tissue [5].

Macrophages play important roles in linking innate with adaptive immunity and their capacity of immune-regulatory, possessing functions including phagocytosis, cytokine production, and antigen presentation [6]. Macrophages are often called tumor-associated macrophages (TAMs) in tumors and are considered as an important component of the tumor microenvironment, featuring remarkable diversity and plasticity [7]. M1 macrophages (classical activation) are mediated by interferon-gama (IFN-γ) and lipopolysaccharide (LPS), while M2 macrophages (alternative activation) are induced by interleukin (IL)-4, IL-10, and IL-13 [8]. M2 macrophages produce high levels of IL-10, express scavenger receptors, and exhibit tissue repair and anti-inflammatory functions [9, 10]. In contrast, M1 macrophages are potent killers of pathogens and tumor cells, generating pro-inflammatory cytokines and expressing high levels of MHC molecules [9].

Nuclear factor (NF)-κB plays an important roles in inflammation and immunity, which is a generic term for a family of transcription factors [11]. Various studies have indicated that inhibition of NF-κB activation helps to drive the tumor-promoting phenotype of TAMs [12, 13]. Macrophages are polarized to M2-like phenotype by malignant epithelial cells [14]. Positive correlation was found between macrophage infiltrated in the tumor stroma and OSCC with a higher histological grading [15]. Ni et al demonstrated that TAM also played a unique role in prognosis of patients with OSCC [16]. Besides, study found that the NF-κB pathway might involve in the induction of M2 phenotype macrophage polarization by OSCC cells [17]. In addition, macrophage polarization influences progression and survival of solid malignancies, including OSCC [18, 19].

Signal regulatory protein α (SIRPα) is a cell-surface protein mainly expressed on myeloid cells, including macrophages and dendritic cells [20]. SIRPα may play an important role in immune regulation because the extracellular region of SIRPα is heavily glycosylated and consists of three immunoglobin superfamily (IgSF) domains, which are similar to the T cell receptor (TCR) [21]. SIRPα can bind to either widely expressed transmembrane ligand CD47 or soluble ligands [21]. The expression of SIRPα and CD47 are varies during infection and malignancies, and they are involved in the pathogenesis of various tumor, such as melanoma, leukaemia, lung cancer [22, 23]. Our previous study showed that CD47 was up-regulated in oral leukoplakia (OLK) and OSCC, suggesting cancer cells may evade phagocytosis of macrophages through the interaction of CD47 with SIRPα.

However, the profile of SIRPα expression in OLK and OSCC and the mechanism by SIRPα regulating macrophages or oral cancer cells remain unclear. Thus, the aim of the current study was to investigate the distribution and the incidence of SIRPα on macrophages and the relation to clinicopathological factors. Moreover, this study explored the regulation of SIRPα on cell phagocytosis, phenotype, proliferation, invasion, and migration of macrophages. At the last, the relationship between SIRPα and NF-κB was studied at regulating the phenotype of macrophages.

## RESULTS

### The expression of SIRPα in OLK and OSCC specimens

**Figure 1:**
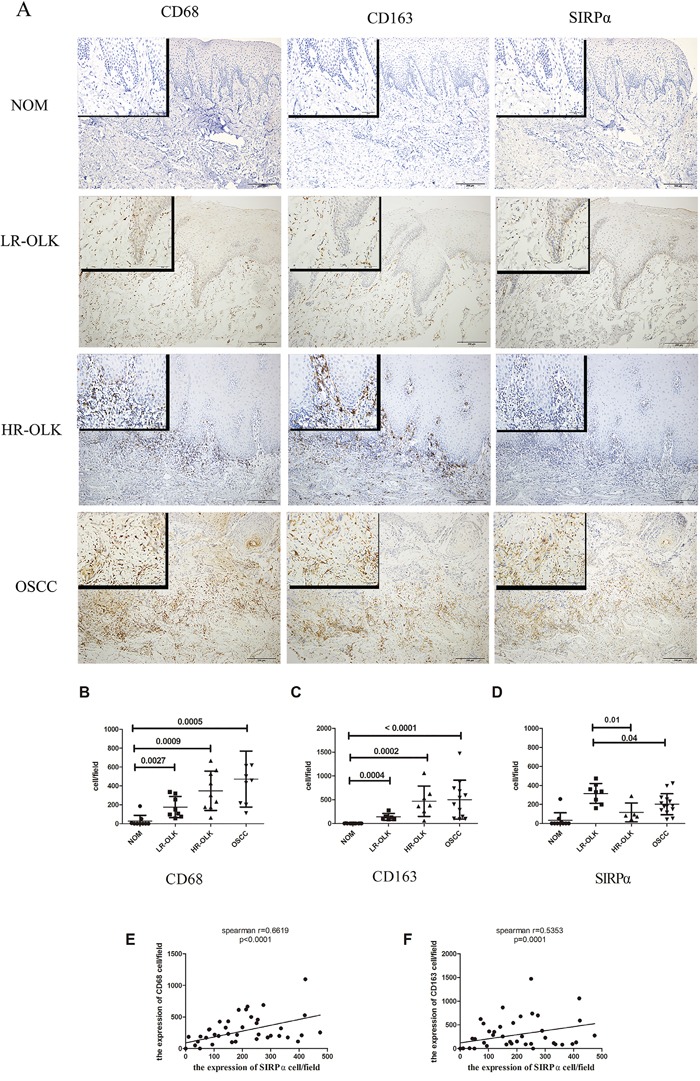
The expression of CD68, CD163, and SIRPα in NOM, LR-OLK, HR-OLK, and OSCC specimens (×100) (×400) **A.** The number of expression of CD68 **B.** and CD163 **C.** were increased from NOM to OSCC, along with the rising of histological grades of dysplasia. The expression of SIRPα was higher in LR-OLK than HR-OLK and OSCC **D.** Spearman's rank analysis was used to analysis the correlation between CD68, CD163, and SIRPα **E, F.** Data are presented as mean ± SD, One way ANOVA with post Tukey test.

SIRPα was expressed in the cytoplasm and frequently observed in OLK and OSCC samples (Figure 1A). Few cells were positively stained in NOM. The expression of SIRPα in LR-OLK, HR-OLK, and OSCC were increased comparing with NOM (*p*<0.05). The expression of SIRPα in LR-OLK was higher than OSCC (*p*=0.04) (Figure 1D). Compared with OSCC, the expression of SIRPα in HR-OLK was lower than OSCC, but there was no significant difference (*p*=0.15).

### Correlation between macrophages and SIRPα in OLK and OSCC

To investigate the expression of SIRPα on macrophages in OLK and OSCC, the expression of two macrophage markers, CD68 (macrophages) and CD163 (M2 macrophages) were used to identify the precise location of SIRPα. The majority of the infiltrated macrophages were located in the subepithelial stroma. CD163-positive and CD68-positive macrophages were rarely detected in the epithelial stroma of the NOM specimens. Although the expression of CD163-positive macrophages was slightly lower than that of CD68-positive macrophages, the difference was not significant (*p*>0.05). Both the expression of CD68 and CD163 were detected increased significantly by pathological grade (Figure 1B, 1C). The expression of CD68 and CD163 in LR-OLK (*p*<0.05), HR-OLK (*p*<0.05), and OSCC (*p*=0.0005) were gradually increased as compared with that in NOM. In addition, the expression of CD68 on macrophages positively correlated with the expression of SIRPα, and the expression of CD163 on macrophages negatively correlated with the expression of SIRPα (*p*< 0.0001, *p*=0.0001; Figure 1E, 1F).

**Figure 2:**
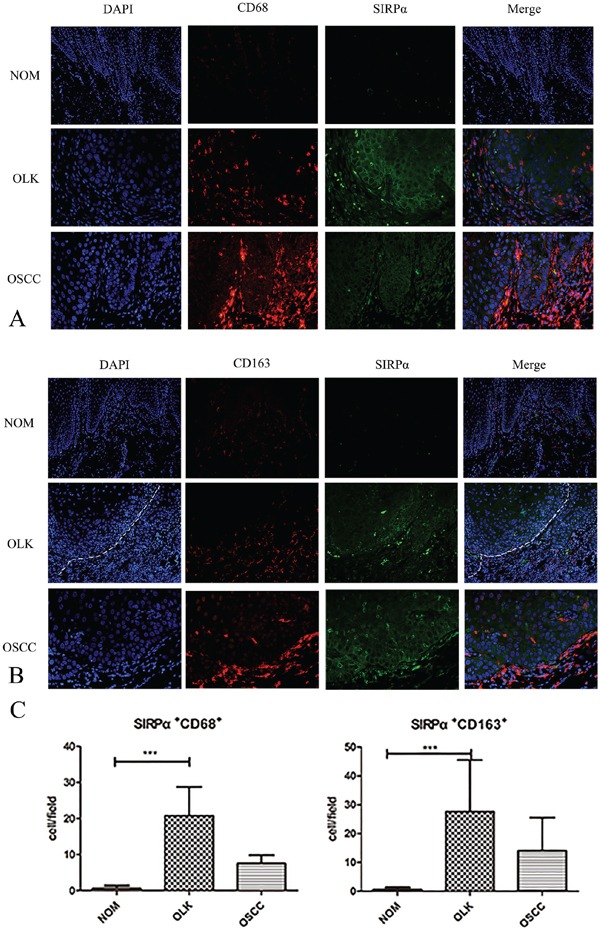
The expression of SIRPα, CD68, and CD163 in OLK and OSCC sample **A.** Double-labeling immunofluorescence for CD68 and SIRPα in OLK and OSCC samples. **B.** Double-labeling immunofluorescence for CD163 and SIRPα in OLK and OSCC samples. **C.** The infiltrated cells of SIRPα^+^CD68^+^ and SIRPα^+^CD163^+^ in OLK were higher than NOM and OSCC. Data are presented as mean ± SD, One way ANOVA with Tukey test. *p < 0.05, **p < 0.01, and ***p < 0.001.

To further characterize the expression of SIRPα on macrophages, we used double-labeling immunofluorescence to detect the coexpression of CD68, CD163, and SIRPα. The expression of SIRPα was mainly located in the subepithelial lesion in OLK, and in OSCC was infiltrated in epithelial lesion. CD163 was expressed in the subepithelial lesion, and CD163 cells mainly located under the basal lamina co-localized with SIRPα (Figure 2A, 2B). The percentages of CD163-positive macrophages and SIRPα-positive cells were increased in OLK than NOM (*p*<0.01). The percentages of CD68-positve macrophages in OLK co-expressed with SIRPα were significant higher than NOM (*p*<0.05; Figure 2C). The percentages of CD163-positive macrophages or CD68-positive macrophages co-expressed with SIRPα were increased in OLK than OSCC (*p*<0.05).

### Conditioned media of OSCC cell lines reduced the expression of SIRPα on macrophages

**Figure 3:**
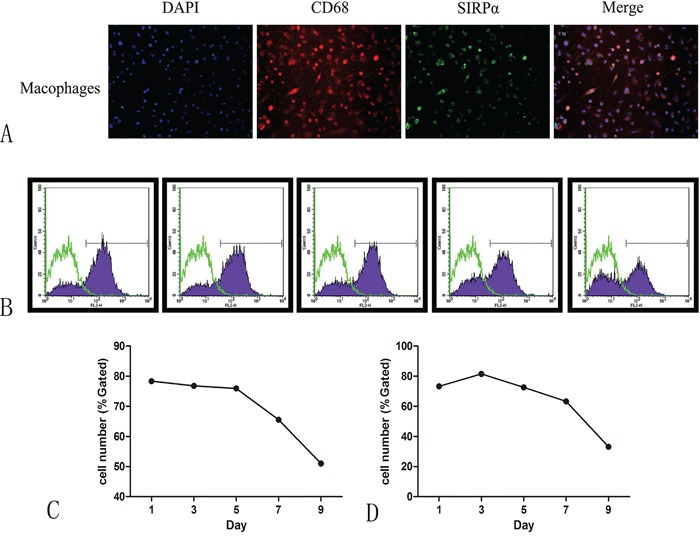
The expression of SIRPα on macrophages after co-culture with the supernatant of oral cancer **A.** CD68 were co-expressed with SIRPα on macrophages after co-cultured with Cal-27 or SCC-9. **B.** The expression of SIRPα was decreased gradually when macrophages were co-cultured with supernatant of SCC-9 **C.** or Cal-27 **D.** cells for 1, 3, 5, 7, and 9 days.

The co-expression of CD68 and SIRPα was examined using double-labeling immunofluorescence to detect the sub-cellular expression of SIRPα on macrophages. PMA stimulated THP-1 cells into macrophages. Macrophages cultured with the conditioned media of Cal-27 cells after stimulation. CD68 and SIRPα expression were found on the cytoplasm of macrophages (Figure 3A).

Flow cytometric analysis was applied to study the effect of cancer cells on the expression of SIRPα on macrophages. THP-1 cells were cultured with conditioned media of Cal-27 or SCC-9 cell lines for 0, 1, 3, 5, 7, and 9 days after stimulated by PMA. The expression of SIRPα was decreased gradually, manifesting time-dependent pattern (Figure 3B, 3C).

### Cal-27 cell lines promoted the production of M2-polarized macrophages cells

**Figure 4:**
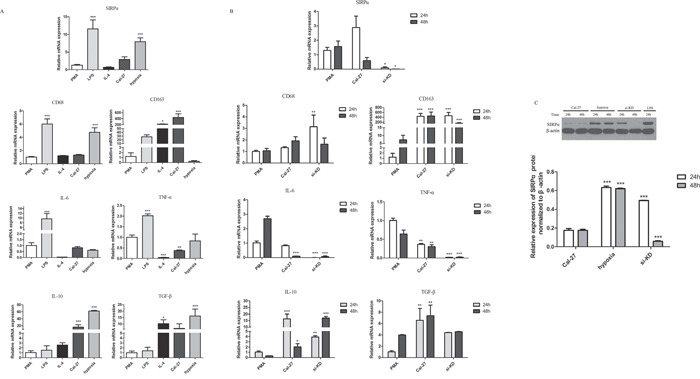
Cytokine profiles and the expression of SIRPα, CD68, and CD163 on macrophages THP-1 cells were stimulated by different factors after induced by PMA into macrophages. **A.** Macrophages stimulated by LPS (1 μg/mL) for 24 h expressed high SIRPα, CD68, IL-6, and TNF-α, and displayed low IL-10 and TGF-β. In IL-4 group, macrophages decreased the expression of SIRPα, CD68, IL-6, TNF-α while increased the expression of CD68, CD163, and TGF-β after treated with 10 μg/mL for 24 h. Macrophages under hypoxia for 24 h increased the production of SIRPα, CD68, IL-10, and TGF-β, while decreased the production of IL-6 and TNF-α. **B.** Macrophages were co-cultured with Cal-27 cells after knockdown of SIRPα. In si-KD group, macrophages displayed low SIRPα, IL-6, TNF-α, and high CD68, CD163, IL-10, and TGF-β. **C.** The protein expression of SIRPα in si-KD group was increased than Cal-27 group in 24 h but decreased significant in 48 h co-culture. Analysis via one-way ANOVA with Tukey test. *p < 0.05, **p < 0.01, and ***p < 0.001 versus PMA group.

To determine the effect of oral cancer cell lines on PMA-treated THP-1 cells, we induced the TPH-1 cells into macrophages with PMA for the first 24 h and co-cultured them with Cal-27 cell lines in transwell plate for another 24 h, 48 h. The treated THP-1 cells underwent morphological changes characterized by increased size and improved adherence, implying their differentiation from monocytes into macrophages. As shown in Figure 4A, the cells treated with PMA only had high TNF-α, and IL-6 levels and low TGF-β level. By contrast, comparing with PMA group, Cal-27 group elevated the expression of CD163, IL-10, and TGF-β mRNA. The expression of IL-10 was increased dramatically in 24 h and increased slightly after 48 h co-culture (*p*<0.05). Furthermore, the expression of IL-6, and TNF-α mRNA were decreased. These results indicated that in the co-cultured system, macrophages were induced to M2 phenotype.

Cells were also treated with IL-4/LPS to set as the positive controls. After THP-1 cells were induced by PMA and stimulated with LPS, the expression of CD68 protein in macrophages was up-regulated and the expression of CD163 protein was decreased comparing with Cal-27 group (Figure 4A). The cells in IL-4 group were similar to M2-polarized macrophages in terms of low TNF-α, and IL-6 levels and high CD68, CD163, and TGF-β levels. Also, under hypoxic environment, comparing with Cal-27 group, the expression of CD68, IL-10, and TGF-β mRNA were increased in hypoxia group and the expression of CD163 mRNA was decreased (Figure 4A).

### SIRPα was associated with the phenotype of macrophages switch

In the co-cultured system, the expression of SIRPα mRNA and protein on macrophages was slightly increased in Cal-27 group than PMA group in 24 h. After 48 h co-cultured with Cal-27 cells, the expression of SIRPα mRNA was decreased dramatically (Figure 4C, Figure 4A). To address whether SIRPα regulates the phenotype switch of macrophage, SIRPα expression in THP-1 was inhibited by siRNA transfection (si-KD). Compared with the Cal-27 group, the expression of SIRPα in si-KD group was decreased after co-cultured (Figure 4C). We further compared the mRNA expression of M1-related and M2-related genes of macrophages in Cal-27 control group and si-KD group. In si-KD group, the expression of IL-10, TGF-β were elevated, and the production of IL-6, TNF-α were down-regulated (Figure 4B). Moreover, the expression of CD68, CD163 mRNA were increased significantly in si-KD group.

Furthermore, under hypoxic environment, both protein and mRNA expressions of SIRPα on macrophages in hypoxia group were up-regulated than Cal-27 group, while slightly decreased than LPS group (*p*<0.05) (Figure 4C, Figure 4A). In LPS group, the expression of SIRPα protein was increased than Cal-27 group (*p*<0.05). Stimulated by IL-4, macrophages expressed less SIRPα than macrophages in LPS and PMA groups (*p*<0.05) (Figure 4A). These results demonstrated that these components existing in oral cancer microenvironment may influence SIRPα expression on macrophages.

### SIRPα negatively regulated the proliferation of macrophages

**Figure 5:**
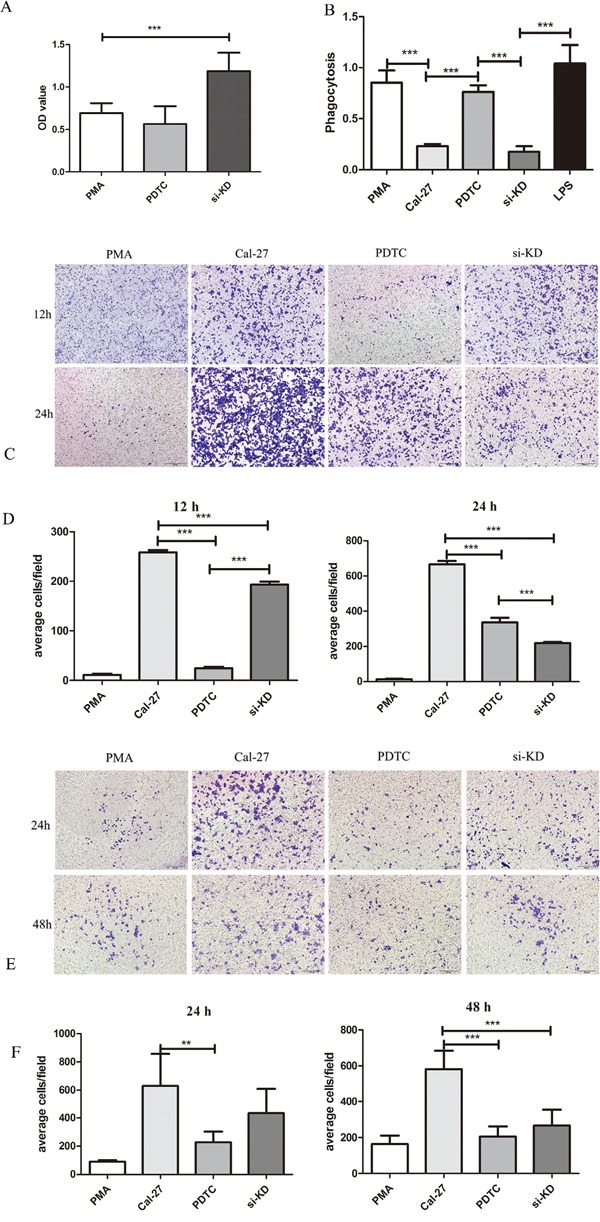
SIRPα and NF-κB regulated the proliferation, phagocytosis, migration, and invasion of macrophages THP-1 cells were stimulated by LPS, siRNA, and PDTC after induced by PMA into macrophages. After stimulation, macrophages were co-culture with Cal-27 cells in Cal-27, si-KD, and PDTC (inhibitor of NF-κB) groups. **A.** the proliferation of macrophages was increased after knockdown of SIRPα. **B.** the phagocytosis ability of macrophage was inhibited in si-KD group while no significant change in PDTC group. **C** and **D.** the migration cells were decreased both in si-KD group and PDTC group. **E** and **F.** the invasion cells were decreased both in si-KD group and PDTC group. Analysis via one-way ANOVA with Tukey test. *p < 0.05, **p < 0.01, and ***p < 0.001.

To investigate the effect of SIRPα on the proliferation of macrophages, CCK8 assay was utilized. The proliferation of macrophages in si-KD group were up-regulated than PMA control group (*p*<0.01; Figure 5A).

### SIRPα negatively regulated macrophages recruitment and invasion to OSCC cells

Transwell assay was used to investigate whether SIRPα could regulate macrophages migration and invasion during tumor exposure. In si-KD group, after 24 h and 48 h of co-incubation with tumor cells, the invasive capacity of macrophages was slightly decreased than Cal-27 and significantly increased than PMA group (*p*>0.05, *p*<0.05) (Figure 5E, 5F). The migration ability was significantly increased comparing with PMA group and decreased than Cal-27 group when SIRPα expression on macrophages was silenced (*p*<0.05) (Figure 5C, 5D).

### Knockdown of SIRPα inhibited phagocytosis ability of macrophages

To evaluate whether SIRPα could influence the phagocytosis ability of macrophages, neutral red uptake assay was utilized. Comparing with PMA control group and LPS group, the phagocytosis ability of macrophages in si-KD group was inhibited significantly (*p*<0.01). The same influence was found in Cal-27 group (*p*<0.01) (Figure 5B).

### SIRPα on macrophages inhibited the recruitment of Cal-27 cells

**Figure 6:**
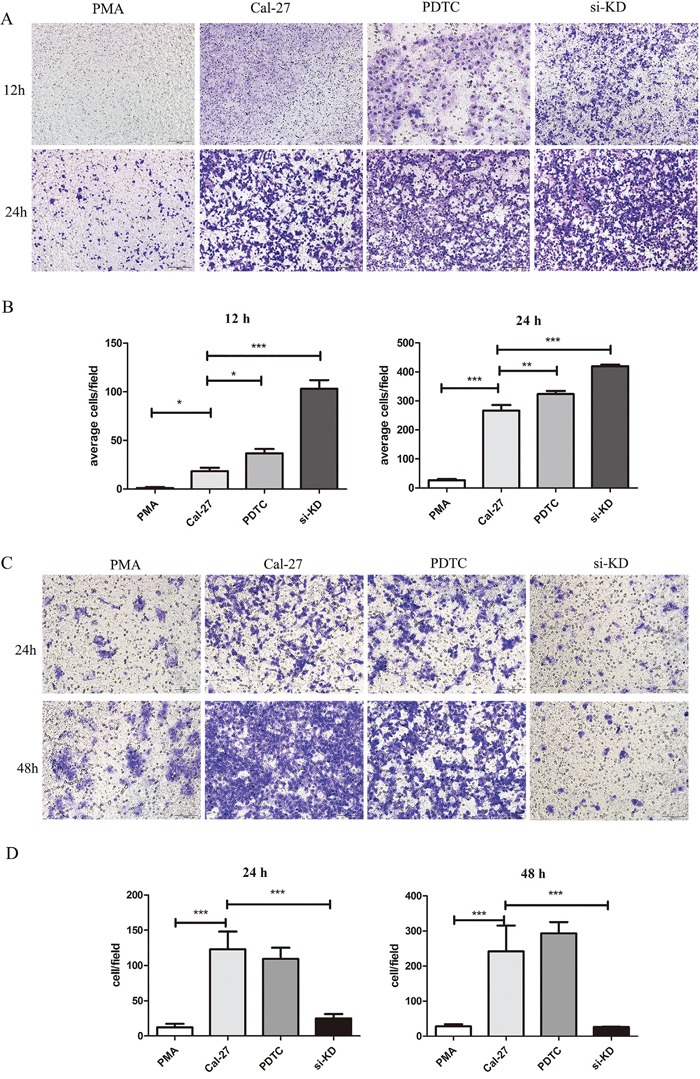
SIRPα and NF-κB regulated the migration and invasion of Cal-27 cells THP-1 cells were stimulated by siRNA and PDTC (inhibitor of NF-κB) after induced by PMA into macrophages. After stimulation, macrophages were co-culture with Cal-27 cells in Cal-27, si-KD, and PDTC groups. **A** and **B.** the migration Cal-27 cells were increased in si-KD group and PDTC group. **C** and **D.** the invasion Cal-27 cells were decreased in si-KD group and PDTC group. Analysis via one-way ANOVA with Tukey test. *p < 0.05, **p < 0.01, and ***p < 0.001.

Since SIRPα significantly inhibited the macrophages recruitment and invasion to Cal-27 cells, we then investigated the influence of SIRPα on Cal-27 cells. After knockdown of SIRPα in macrophages, the migration of Cal-27 cells were increased in si-KD group than Cal-27 group (*p*<0.05) (Figure 6A, 6B). At the meantime, the invasion of Cal-27 cells was decreased in si-KD group than Cal-27 and PMA groups (*p*<0.05) (Figure 6C, 6D).

### SIRPα was regulated by NF-κB signaling pathway

**Figure 7:**
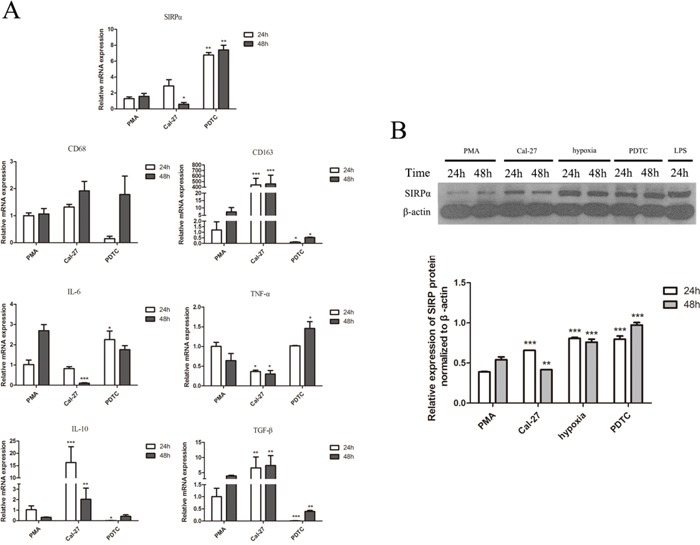
The effect of NF-κB on the expression of cytokine profiles, CD68, CD163, and SIRPα on macrophages THP-1 cells were stimulated by LPS, hypoxia, and PDTC (inhibitor of NF-κB) after induced by PMA into macrophages. After stimulation, macrophages were co-culture with Cal-27 cells for 24 h and 48 h in Cal-27, hypoxia, and PDTC groups. **A.** Macrophages co-cultured with Cal-27 cells for 24h and 48 h after PDTC (50 μmol/mL) inhibited NF-κB in macrophages. Macrophages in PDTC group presented high SIRPα, IL-6, TNF-α, and low CD68, CD163, IL-10, and TGF-β. **B.** The expression of SIRPα in Cal-27 group was increased than PMA group. In PDTC group, the expression of SIRPα was increased than Cal-27 group after inhibiting NF-κB. The expression of SIRPα in LPS and hypoxia groups were increased. Analysis via one-way ANOVA with Tukey test. *p < 0.05, **p < 0.01, and ***p < 0.001 versus PMA group.

NF-κB is considered as important transcription factor in macrophages linking inflammation and cancer, the study explored whether NF-κB could affect the expression of SIRPα and the polarization of macrophages [24]. The expression of SIRPα was analyzed with western blot to determine the role of NF-κB during polarization of THP-1 cells. Comparing with Cal-27 group, the expression of SIRPα protein was inhibited in si-KD group (Figure 4C). From another direction, after using PDTC (pyrrolidine dithiocarbamate ammonium) to inhibit the activation of NF-κB, the expression of SIRPα protein was up-regulated than Cal-27 group (Figure 7B). Moreover, in PDTC group the macrophages were switch into M1 phenotype with the elevated IL-6 and TNF-α mRNA levels, and reduced CD163, IL-10 and TGF-β mRNA levels (Figure 7A).

### NF-κB regulated macrophages phagocytosis, recruitment and invasion abilities

To determine whether the inhibitor of NF-κB has an effect on the proliferation, phagocytosis ability, migration and invasion of macrophages, CCK-8, co-culture assay and transwell assay were performed. Compared with PMA group, macrophages in PDTC group were present no significant inhibition in proliferation (Figure 5A). The phagocytosis ability of macrophage was enhanced in PDTC group than Cal-27 group (*p*<0.01) (Figure 5B). After stimulated by PDTC, the migratory ability of macrophages were significantly inhibited than in Cal-27 group (Figure 5C, 5D). The transwell invasion assay also showed that the number of invasive cells stimulated by PDTC were significantly lower compared with the Cal-27 group both in 24 h and 48 h (*p*=0.0081, *p*=0.004) (Figure 5E, 5F).

### NF-κB regulated recruitment and invasion abilities of Cal-27 cells

The influence of NF-κB in macrophages on the recruitment and invasion abilities of Cal-27 cells was further explored. Cal-27 cells co-cultured with macrophages in PDTC group and the migration cells were increased than PMA group in 12 h and 24 h (*p*<0.05, *p*<0.01) (Figure 6A, 6B). In invasion experiment that invaded Cal-27 cells in PDTC group were increased (*p*<0.05) (Figure 6C, 6D).

## DISCUSSION

In the tumor microenvironment, macrophages comprise a major population of tumor-infiltrating immune cells [25]. Macrophages exhibit either pro- or anti-tumor properties depending on their phenotype [25]. In this study, we detected the expression of SIRPα in OLK and OSCC, and the correlation with macrophages. In exploring the effect of SIRPα on macrophages in OSCC, we found that SIRPα was down-regulated on macrophages when co-cultured with OSCC, and increased the phagocytosis ability of macrophages. Furthermore, it negatively regulated migration and invasion of macrophages to OSCC. At the meantime, our data demonstrated that SIRPα was associated with the phenotype switch of macrophages through NF-κB pathway and played a negative role in tumor migration.

TAMs is a predominant cellular component in tumor microenvironment, promoting the progression, angiogenesis, and metastasis in various tumors [26, 27]. The present study found that in OLK and OSCC, the expressions of CD163^+^ macrophages was higher than that in normal oral mucosa, demonstrating the important role of M2 macrophages in the development of OLK and OSCC [28]. Besides, Mori et al. proved that the expressions of CD163^+^ cells were significantly increased along with the pathological grade in OSCC and concluded that the infiltrated TAMs have an M2 phenotype in OSCC [29]. In addition, there was a study announcing that the CD163^+^ macrophages in OLK appear to possess an M1 phenotype [30]. Pan et al. found that in the tumor tissues, macrophages present an immunosuppressive phenotype like M2. However, a larger amount of M1-like cells are contained in the peritumor tissues [24]. It might suppose that macrophages were educated by tumor cells from M1 to M2 phenotype, and this process was along with the progression of OLK and OSCC. SIRPα are members of glycoprotein expressed on macrophages, promoting immune recognition and priming phagocytosis of pathogens or malignant cell [31]. The present study found the expression of SIRPα in LR-OLK was higher than OSCC. Moreover, the expression of SIRPα was associated with macrophages. In accordance with this, gradually decreased expression of SIRPα on macrophages was appeared in vitro co-culture assay. These results might suggesting that SIRPα could plays a vital role in the progression of OLK and OSCC, meanwhile, SIRPα could be co-marker with macrophages to prognosis the OLK and OSCC.

SIRPα-CD47 interaction is served as a “don't eat me” and “self-recognized” signaling and it could be a therapeutic target for human tumors [23, 32–34]. When tumor cells treated with anti-CD47 antibodies, macrophages enhanced the phagocytic capability against tumor cells [35]. Considering the relatively limited tissue expression, SIRPα may be a better target compared with agents targeting CD47 which is ubiquitously expressed [23]. In this study, we observed that macrophages co-cultured with OSCC cells expressed lower level of SIRPα compared with control group. Meanwhile, in SIRPα knockdown macrophages, the phagocytosis ability of macrophages were inhibited. Thus, it demonstrated that OSCC might inhibit the phagocytosis ability of macrophages through down-regulated the expression of SIRPα. Many studies have shown that the invasive and migratory behavior of malignant cells could be increased by macrophages [36]. The current data represented that relative lower expression of SIRPα on macrophages in co-culture assay was correlated with the recruitment and invasion of oral cancer cells, indicating that oral cancer might exploit the macrophages with SIRPα reduction to profit themselves. Interestingly, even without cell-cell direct interaction, knockdown of SIRPα on macrophages could increase the migration of Cal-27 cells, suggesting that the substance released by macrophages may play a critical part in tumor migration.

Macrophages can be differentiated into M1- or M2-like macrophages, and then different phenotype cells produce Th1 or Th2 cytokines [7]. M1 macrophages produce several pro-inflammatory cytokines, such as TNF-α and IL-6, and the M2-like phenotype of TAM increase the expression of immunosuppressive cytokines such as TGF-β and IL-10. [5]. Importantly, IL-10 is an immunosuppressive cytokine and lead to M2 macrophage differentiation [37]. Our data indicated that macrophages produced high level of IL-10 involving in the macrophages differentiation in the first 24 h co-culture and kept the production of IL-10 in a stable level. In addition to activating macrophages, in the tumor environment, TNF-α is able to promote angiogenesis [38]. A significant increase was observed in the production of Th1 cytokines by the differentiated M1 macrophages stimulated by LPS. Meanwhile, stimulated by IL-4, macrophages increased the secretion of Th2 cytokines. Our study further showed that pro-inflammatory cytokines genes were inhibited when knockdown the expression of SIRPα, along with the adjustment of macrophages immune status, suggesting that SIRPα might play a possible role in regulating macrophages phenotype switch. However, it was hard to elaborate why the protein of SIRPα was increased when macrophages co-cultured with Cal-27. We speculated that macrophages would switch to M1 phenotype before being re-educated to M2 macrophages by cancers. Mori et al. indicated that a Th1-dominated microenvironment was formed in OLK lesions and suggested that Th1-derived IFN-γ could be able to polarize the infiltrated macrophages switch to the M1 phenotype [30]. However, in premalignant lesions, alteration in the phenotypes and features of TAMs during tumor development have not been characterized. Thus, further investigation is needed to explore the precise role of SIRPα on macrophages under premalignant microenvironment.

Various studies have demonstrated that inhibition of NF-B activation helps to drive the tumor-promoting phenotype of TAMs to antitumor phenotype [13, 39]. Redirecting M2-like TAM toward M1-like phenotype by reactivating NF-κB induced significant antitumor immune response in mammary carcinoma [40]. Besides, TAM increased their tumoricidal activity after targeted deletion or inhibition of IKKβ [40]. In spite of the fact that NF-κB is viewed as a noteworthy pro-inflammatory transcription factor, recent studies have demonstrated that NF-κB activation also controls anti-inflammatory pathways, especially in macrophages [41]. When NF-κB signaling is repressed particularly in TAMs, they get to cytotoxic to tumor cells and switch to a “classically” activated phenotype [9]. PDTC, as an inhibitor of NF-κB pathway, could enhance macrophages differentiation into pro-inflammatory M1 phenotype [42]. In our study, PDTC up-regulated the expression of SIRPα and influenced the polarization of macrophages. Moreover, overexpression of SIRPα was reported to negatively modulate nuclear factor NF-κB signaling [23]. Inhibition of NF-κB pathway promotes M1-like phenotype in TAMs considering that improved tumoricidal activity is connected with M1 characteristics [43, 44]. Since NF-κB is a vital transcription factor in immune response against tumor, this result might uncover that the tumoricidal activity of SIRPα-KD macrophages may be diminished as well. Therefore, SIRPα might regulate NF-κB signaling to switch the phenotype of macrophages, which could be a novel target by increasing macrophages tumoricidal activity.

## MATERIALS AND METHODS

### Patients and samples

Incisional biopsy samples from 20 clinically diagnosed OLK (n=10) and OSCC (n=10) were sent for histological confirmation according to the classical histopathological features. Five normal oral mucosa (NOM) samples obtained during third molar removal were used as negative controls in this study. All samples were obtained from the Department of Oral Medicine, School and Hospital of Stomatology, Wuhan University. For subgroup analysis, OLK samples were divided into OLK with low-risk dysplasia (LR-OLK) and OLK with high-risk dysplasia (HR-OLK) according to the scheme of the World Health Organization (2005) [45]. The characteristics of patients were showed in Table 1. Informed consent was obtained from each patient and normal control, and whole procedures were approved by the Ethical Committee of Hospital of Stomatology, Wuhan University.

**Table 1:** The characteristics of patients

Sample	Gender	Age	Location	Pathological grade	Tobacco	Alcohol
K1	M	60	tongue	HR-OLK	N	N
K2	M	57	tongue	HR-OLK	N	Y
K3	F	58	tongue	HR-OLK	N	N
K4	M	48	bucca	LR-OLK	Y	Y
K5	M	47	bucca	HR-OLK	N	Y
K6	M	75	tongue	HR-OLK	N	N
K7	F	71	tongue	LR-OLK	N	N
K8	M	75	bucca	LR-OLK	N	N
K9	M	48	tongue	LR-OLK	N	Y
K10	F	45	tongue	LR-OLK	N	N
C1	F	60	tongue	OSCC	N	N
C2	M	46	tongue	OSCC	Y	N
C3	F	45	tongue	OSCC	N	N
C4	F	57	bucca	OSCC	N	N
C5	M	75	lip	OSCC	N	N
C6	F	33	tongue	OSCC	N	N
C7	M	76	lip	OSCC	N	N
C8	M	48	tongue	OSCC	Y	Y
C9	F	78	bucca	OSCC	N	N
C10	M	50	bucca	OSCC	N	Y
N1	F	45	gingiva	NOM	N	N
N2	M	23	gingiva	NOM	N	N
N3	F	53	lip	NOM	Y	N
N4	F	16	lip	NOM	N	N
N5	F	56	lip	NOM	N	N
N6	M	18	bucca	NOM	N	N
N7	M	61	tongue	NOM	N	N
N8	F	49	lip	NOM	N	N
N9	F	21	tongue	NOM	N	N
N10	M	40	lip	NOM	N	Y

Gender F: female; Gender M: man; OLK: oral leukoplakia; OSCC: oral squamous cells carcinoma; NOM: normal oral mucosa; N: no; Y: yes.

### Immunohistochemistry

Concentrations of primary antibodies were used for monoclonal mouse anti-human CD68 1:400, monoclonal mouse anti-human CD163 1:400, and for polyclonal rabbit anti-human SIRPα 1:50, and all were obtained from Abcam biotechnology. Paraffin sections were dewaxed and rehydrated through a series of xylene, graded alcohol and water immersion steps. The deparaffinized and hydrated slides were treated with 3 % H_2_O_2_ at 37 °C for 20 min to block endogenous peroxidase activity and then washed with phosphate buffered saline (PBS). Slides were treated with 5 % normal goat serum for 30 min to block non-specific binding, and then incubated with primary antibodies at 4 °C overnight as suggested by the product instruction. After washing with PBS, they were incubated with biotinylated goat anti-mouse or goat anti-rabbit IgG antibody (SP Kit; Zhongshan Golden Bridge Ltd., Beijing, China) for 20 min and washed again with PBS. They were treated with 3’3-diaminobenzidine tetrachloride (DAB) for 3 min, and counter-stained with Mayer's hematoxylin, dehydrated and mounted with a glass coverslip and xylene-based mountant. Negative controls were incubated with PBS instead of primary antibodies.

### Double-labelling immunofluorescence

Double-labelling immunofluorescence was used to define expressing of SIRPα in OLK and OSCC. Five control, five OLK and five OSCC specimens were randomly selected from the previously cut sections and were labelled with a primary SIRPα antibody (as above) and anti-CD163 antibody or anti-CD68 antibody. The secondary antibodies were Alexa Fluor 488 goat anti-mouse IgG and Alexa Fluor 596 goat anti-rabbit IgG (Zhongshan Golden Bridge Ltd., Beijing, China), and both used at diluted solution 1:100 for 60 min at room temperature.

### Evaluation and cell counting

Slides were interpreted by two investigators who were blind to clinical details. Cell cytoplasm and membrane localization of SIRPα, CD68, and CD163 were considered as positive results. In each slide, three staining areas throughout the epithelium were selected randomly, and positive cells were counted in high-power fields (Olympus Optical Co Ltd., Tokyo, Japan). Percentage of positive cells per field of SIRPα, CD68 or CD163 was calculated and the mean percentage of positive cells per field of each case was represented by the average of three areas of each slide.

### Cell line culture

THP-1 cells are a human leukemia monocytic cell line extensively used to study the modulation of monocytes and macrophages [46]. THP-1 cells in the monocyte state can be differentiated into a macrophage-like phenotype stimulated by phorbol-12-myristate-13-acetate (PMA) [47]. THP-1 cells were cultured in RPMI 1640 medium (Invitrogen, Carlsbad, CA, USA) supplemented with 10 % FBS, 100 units/ml, and 100 ng/ml streptomycin. Cal-27 cell line was cultured in DMEM supplemented, and SCC9 cell line was cultured in DMEM F-12, all with 10 % bovine serum and 1 % gentamycin. All cell lines were incubated at 37 °C under a humidified 5 % CO_2_ and 95 % air atmosphere. The conditioned media of Cal-27 and SCC9 cell lines were extracted and stored at 4 °C after cell line was grown until 89-90 % confluent. The cells were free of mycoplasma contaminated during the study period until now.

### Cells immunofluorescence

To quantitate the effect of cancer cells on macrophage, an in vitro indirect co-culture system was employed. Briefly, 2×10^5^ THP-1 cells/ml RPMI were seeded in 6-well plates and stimulated by 100 nM PMA for 24 h, to induce the differentiation of cell into macrophages. After washed by PBS for three times, the conditioned media of Cal-27 and SCC9 cell lines were added to each well, and incubated for 1 day. Cells were stained by using rabbit polyclonal antibody to SIRPα and mouse monoclonal antibody to CD68 (Abcam) at appropriate dilutions, followed by incubation with Cy3-conjugated anti-rabbit IgG antibodies and Cy5-conjugated anti-mouse IgG antibodies (Zhongshan Golden Bridge Ltd., Beijing, China), and observed by a fluorescent microscope (Leica Microsystems, German).

### Flow cytometric analysis

1 × 10^6^ THP-1 cells/well in triplicates were placed in wells of two plates. Cells were stimulated with PMA for 24 h. After washed by PBS for three times, conditioned media of Cal-27 and SCC-9 cell lines were added and incubated for 0, 1, 3, 5, 7, 9 days, respectively. After cultured day, the supernatant was discarded and the cells were washed twice in PBS. The cells were resuspended in 100 μL of PBS with SIRPα antibody and CD163 antibody or with a control in PBS containing 0.5 % (w/v) BSA, and were incubated for 30 min at 4 °C. Cells were analyzed on a fluorescent activated cell sorter (BD Biosciences, San Jose, CA). APC-CD163 and PE-SIRPα antibodies were used and purchased from Biolegend (San Diego, CA). After incubation, the tubes were centrifuged at 300 ×g at 37 °C for 5 min and washed three times in PBS with centrifugation at 300 ×g at 37 °C for 5 min between each wash. PBS from the third wash was left in the tubes for flow cytometry. Results were presented using FlowJo (FlowJo LLC, Ashland, USA).

### Co-culture assay

6-well transwell plate with a polycarbonate filter membrane of 0.4 μm pore size (Corning, Acton, MA, USA) was used to establish co-culture system. The upper compartment of the transwell chamber was seeded with THP-1 cells at a density of 1×10^5^ in 200 μl of RPMI 1640 medium, and the lower chamber was seeded with Cal-27 cells at a density of 1×10^4^ in 500 μl of DMED medium.

For subgroup analysis, according to different stimulation, co-culture groups were divided into PMA group, Cal-27 group, si-KD group, PDTC group, LPS group, IL-4 group, and hypoxia group. THP-1 cells in all groups were induced to macrophages by 100 nM PMA. For PMA group, macrophages were co-cultured with DMEM medium. For Cal-27 group, THP-1 cells were co-cultured with Cal-27 cells for 24 h and 48 h after stimulated by PMA. Macrophages in si-KD group were transfected with small interference RNA (siRNA) for 24 h and after transfection, macrophages were co-cultured with Cal-27 for 24 h and 48 h. For LPS group, macrophages were treated with 1 μg/mL LPS for 24 h. For PDTC group, macrophages were treated with 50 μmol/mL PDTC for 24 h and co-cultured with Cal-27 for 24 h and 48 h. For IL-4 group, macrophages were treated with 10 μg/mL IL-4 for 24 h. Co-culture with DMEM medium, macrophages in hypoxia group were incubated in a hypoxia incubator chamber which was flushed with 1 % O_2_, 5 % CO_2_.

### siRNA transfection

In co-culture assay, macrophages were transfected with SIRPα siRNA (sense, 5’-GACACAATGATATCACAT-3’, antisense, 5’-GCCTCAACCGTTACAGAGA-3’) and negative control siRNA according to the manufacturer's siRNA transfection protocol (RiboBio, Guangzhou, China). Phagocytosis, proliferation, invasion, migration assay and co-culture assay were used to detected cells knockdown by siRNA.

### Western blot analysis

Cultured cells were washed immediately with ice-cold PBS and lysed by RIPA buffer containing PMSF (Beyotime Biotechnology, Shanghai, China) and phosphatase inhibitors (Beyotime Biotechnology). Whole cell lysates were collected after centrifugation at 12 000 g for 15 min at 4 °C, and protein concentration in the supernatants was determined by BCA assay kit (Beyotime Biotechnology). Equal amounts of protein (6 μg/lane) were loaded onto 12 % SDS-polyacrylamide gels, subjected to electrophoresis and transferred to PVDF membranes (Millipore Boston, Massachusetts, USA). After blocking with 5 % nonfat milk for 1 h, membranes were incubated overnight at 4 °C with primary antibody anti-SIRPα (1:100; Santa Cruz Biotechnology), anti-p65^S536^, and anti-phosphorylated-p65^S536^ (1:1000; Cell signaling Technology). After three washes with TBST, membranes were then incubated with HRP-labelled secondary antibody for 1 h at room temperature. The protein bands were visualized using ECL reagent, and quantified by densitometry using Image J. β-actin was used as the internal loading control. Western blot experiments were repeated at least three times.

### Cell migration and invasion assay

Cell migration was assessed by using the 24-well transwell plate with a polycarbonate filter membrane of 8 μm pore size (Corning, Acton, MA, USA). The upper compartment of the transwell chamber was seeded with THP-1 cells at a density of 1×10^5^ in 200 μl of serum-free medium, and the lower chamber was seeded with Cal-27 cells at a density of 1×10^4^ in 500 μl of DMED medium. For the cell migration assay, after 12 h and 24 h incubation, the medium was discarded and the filter membrane was fixed with 4 % formaldehyde, stained with 0.1 % crystal violet and examined under an inverted microscope to count migrated cells in 5 different fields. For the cell invasion assay, macrophages were seeded to the upper compartment of BD BioCoat Matrigel chambers (BD Biosciences, San Jose, CA, USA) at a cell density of 1×10^6^ and incubated for 24 h and 48 h. After incubation, the invaded cells on the lower surface were stained with 0.1 % crystal violet and counted in 5 randomly selected fields. To detect the cell migration and invasion of Cal-27 cells, the same experiment was applied and the upper chamber was seeded with Cal-27 cells while the lower chamber was seeded with THP-1 cells.

### RNA isolation and cDNA synthesis

Total RNA from macrophages was extracted using TRIZOL reagent (Invitrogen, Burlington, ON, USA) according to the manufacturer's instructions. Concentration and purity were determined by NanoDrop 1000 spectrophotometer (Thermo Fisher Scientific Inc, Dubuque, USA). RNA (1.0 μg) was used as template for the synthesis of cDNA (20 μl) with Oligo-dT and AMV reverse transcriptase (Takara, Japan).

### Real-time PCR

RT reaction from 1 μg RNA template was performed using PrimeScript™ RT Reagent Kit with gDNA Eraser (Takara, Japan) as manufacturer's instructions. Real-time PCR was done using SYBR® Premix Ex Taq II (Applied Biosystems, Foster City, CA) and detected by ABI-Prism 7900 Sequence Detector (Applied Biosystems, Foster City, CA). The primers used were listed on the Table 2 and synthesized by Sangon Biotech (Sengon, Shanghai, China). The values were calculated applying the 2^-ΔΔCt^ method. All real-time results were expressed as fold changes in mRNA expression compared to the control cells. All results were normalized to the expression of the housekeeping gene GAPDH in the PCR reactions.

**Table 2:** Primer sequence for RT-PCR

Gene	Primer
CD68	FP 5'-TTGGGTGAGGCGGTTCAGCCAT-3'
	RP 5'-GTGCTCTCTGTAACCGTGGGTGT-3'
CD163	FP 5'-ACATAGATCATGCATCTGTCATTTG-3'
	RP 5'-CATTCTCCTTGGAATCTCACTTCTA-3'
TNFα	FP 5'-TCTTCTCGAACCCCGAGTGA-3'
	RP 5'-CCTCTGATGGCACCACCAG-3'
TGFβ	FP 5'-GGGACTATCCACCTGCAAGA-3'
	RP 5'-CCTCCTTGGCGTAGTAGTCG-3'
SIRPα	FP 5'-GCCCACAGGGATGATGTGAA-3'
	RP 5'-TGTGATATCATTTGTGTCCTGTGT-3'
IL-6	FP 5'-TACATCCTCGACGGCATCT-3'
	RP 5'-ACCAGGCAAGTCTCCTCAT-3'
IL-10	FP 5'-TCAGAGAGGGGGTTAGACCTG-3'
	RP 5'-GAGTTGGTCCTGCCAGACTT-3'

### Cell proliferation assay

THP-1 cells were seeded at a density of 2×10^3^ cells per well in 96-well culture plates. The relative cell number of PMA group, PDTC group, and si-KD group were was then detected by cell counting kit-8 (CCK-8) assay (Dojin Laboratory, Kumamoto, Japan) according to the manufacturer's instructions. Briefly, 10 μL of CCK-8 solution was added to each well during the last 2 h of culture at 37 °C, and absorbance in each well was measured at 450 nm using a 96-well multiscanner autoreader (Thermo Fisher Scientific, USA).

### Neutral red uptake assay for macrophage phagocytosis

The phagocytic ability of macrophage was measured by neutral red uptake. Cells in Cal-27, LPS, si-KD, and PDTC groups were cultured for 24 h, 100 μl neutral red solutions was added and incubated for 30 min. The supernatant was discarded and the cells in 96-well plates were washed with PBS twice to remove the neutral red that was not phagocytized by macrophages. Then cell lysate (ethanol and 0.01 % acetic acid at the ratio of 1:1, 100 μl/well) was added to lyse cells. After cells were incubated in room temperature overnight, the optical density at 540 nm was measured by multiscanner autoreader.

### Statistical analysis

Data were analyzed by GraphPad Prism version 5.0 (Graphpad Software Inc., La Jolla, CA, USA). The relationship between clinicopathological parameters and oral dysplasia grade and expression of CD163, CD68, and SIRPα of macrophage were analyzed using the One-Way ANOVA. Comparison of mean values was performed with a Tukey test. Correlations between the various markers were tested using nonparametric Spearman's rank analysis. The One-Way ANOVA were used to evaluate the differences between experimental and control groups.

## CONCLUSION

Our findings provide a new insight into the importance of SIRPα on macrophages in oral leukoplakia and oral cancer progression. In addition, SIRPα could be applied as an important modulator of the phenotype of macrophages in OSCC by targeting NF-κB. Therefore, SIRPα might be a potential target for oral cancer therapy.
